# Comparative Pangenomics of the Mammalian Gut Commensal *Bifidobacterium longum*

**DOI:** 10.3390/microorganisms8010007

**Published:** 2019-12-18

**Authors:** Korin Albert, Asha Rani, David A. Sela

**Affiliations:** 1 Molecular and Cellular Biology Graduate Program, University of Massachusetts, Amherst, MA 01003, USA; korinalbert@umass.edu; 2 Department of Food Science, University of Massachusetts, Amherst, MA 01003, USA; arani@umass.edu; 3 Department of Microbiology and Physiological Systems, University of Massachusetts Medical School, Worcester, MA 01655, USA

**Keywords:** *Bifidobacterium longum*, comparative genomics, microbiome, beneficial microbes

## Abstract

*Bifidobacterium longum* colonizes mammalian gastrointestinal tracts where it could metabolize host-indigestible oligosaccharides. Although *B. longum* strains are currently segregated into three subspecies that reflect common metabolic capacities and genetic similarity, heterogeneity within subspecies suggests that these taxonomic boundaries may not be completely resolved. To address this, the *B. longum* pangenome was analyzed from representative strains isolated from a diverse set of sources. As a result, the *B. longum* pangenome is open and contains almost 17,000 genes, with over 85% of genes found in ≤28 of 191 strains. *B. longum* genomes share a small core gene set of only ~500 genes, or ~3% of the total pangenome. Although the individual *B. longum* subspecies pangenomes share similar relative abundances of clusters of orthologous groups, strains show inter- and intrasubspecies differences with respect to carbohydrate utilization gene content and growth phenotypes.

## 1. Introduction

The genus *Bifidobacterium* contains over 50 species of Gram-positive anaerobes isolated from host-associated environments, including the lower gastrointestinal tract of primates, other animals, and social insects [1]. *Bifidobacterium longum* is a common colonizer of the human and nonhuman gut and is associated with potential beneficial properties including reduction of gastrointestinal inflammation and pathogen exclusion [2,3,4,5]. *B. longum* strains are assigned to three subspecies, *longum sensu stricto*, *infantis*, and *suis* and colonize adults, infants, and animals, respectively. Notably, subspecies *longum* strains are isolated from infants as well [6,7,8]. Many *B. longum* strains are enriched by host-indigestible dietary carbohydrates, also referred to as prebiotics, which include substrates such as inulin and arabinoxylan [9,10]. Some end products of carbohydrate metabolism, such as the short-chain fatty acid acetate, benefit their host through reducing pathogen-induced colonic epithelial cell death and provide energy to specific tissue types such as the liver [11,12,13]. Thus, *B. longum* subsistence on dietary oligosaccharides is a likely example of host–microbe coevolution, potentially to benefit the population, aggregate microbiome, and host. A noteworthy example of this coevolutionary relationship is human milk oligosaccharide (HMO) metabolism by *B. longum* subsp. *infantis* which is facilitated by a ~40 Kb HMO utilization gene cluster [14,15]. As infants nurse, HMOs reach the colon relatively intact and are utilized as a carbohydrate source by *B. infantis* to thrive in this environment [16,17].

Previous analyses of the *B. longum* pangenome have noted that each subspecies has unique characteristics, including the presence and absence of carbohydrate utilization enzymes [18,19,20]. There are specific and generalizable traits ascribed to each subspecies (e.g., HMO utilization by *B. infantis* strains), although the variability inherent to given subspecies increases as more *B. longum* genomes are sequenced. As more *B. longum* genomes have been sequenced, a more rigorous approach to defining subspecies boundaries may be enabled including the candidate subspecies *suillum*. The current study constructs and investigates the pangenome of 191 *B. longum* strains using Average Nucleotide Identity (ANI) and phylogenetic analytical approaches. The presence/absence of carbohydrate active enzymes and subspecies-specific marker genes are also considered for their utility in defining subspecies boundaries. Finally, in vitro growth on carbohydrate substrates is used to confirm subspecies phenotypic definitions.

## 2. Materials and Methods

### 2.1. Bifidobacterial Propagation and Isolation

Bifidobacteria were routinely propagated on de Man Rogosa Sharpe media (MRS; BD Difco) with 0.05% (*w*/*v*) of L-cysteine (Sigma Aldrich, St. Louis, MO, USA). Cultures were incubated at 37 °C in an anaerobic chamber maintained with a gas mix of 7% H_2_, 10% CO_2_, and N_2_ to balance (Coy Laboratory Products, Grass Lake, MI, USA). Genomic DNA was extracted from liquid cultures using the MasterPure Gram Positive DNA Purification Kit according to the manufacturer’s protocol (Epicentre, Madison, WI, USA). In order to isolate bifidobacteria, fresh feces were mixed into 5 mL of peptone water. Serial dilutions of 10x and 100x were performed on bifidobacterial-specific media agar plates which consisted of MRS, 0.05% (*w*/*v*) L-cysteine, and 0.05% (*w*/*v*) mupirocin (AppliChem Panreac, Chicago, IL, USA). Isolated colonies were identified via the bifidobacteria-specific colorimetric fructose-6-phosphoketolase assay and PCR of the 16s and ITS rRNA gene regions (PCR primers: F-GGTGTGAAAGTCCATCGCT, R-GTCTGCCAAGGCATCCACCA; Sanger sequencing primers: F-GGTGTGAAAGTCCATCGCT, R-CATGCCCCTACGTCCAG) according to Turroni et al. and Milani et al. [21,22,23]. 

### 2.2. Whole Genome Sequencing

DNA quality and quantity were determined using a NanoDrop 2000 Spectrophotometer (Thermo Fisher Scientific, Waltham, MA, USA) and a Qubit 2.0 Fluorometer (Thermo Fisher Scientific, Waltham, MA, USA), respectively. Sequencing libraries were prepared using the NEBNext Ultra II library preparation kit (New England Biolabs, Ipswich, MA, USA). Subsequently, whole-genome sequencing was performed on the Illumina NextSeq platform with v2 reagents to generate 2 × 75 bp paired-end reads (Illumina, San Diego, CA, USA). Reads were assembled de novo via Unicycler v0.4.7 with default parameters, which utilizes SPAdes for the short-read assembly with Pilon, Bowtie2, and Samtools for genome polishing [24,25,26,27]. Assembly graphs were visualized with Bandage [28]. Gene model predictions and annotations were performed using the Rapid Annotation using Subsystem Technology (RAST) annotation service and PROKKA v.1.13 using default parameters [29,30,31]. The Whole Genome Shotgun projects for strains UMA3015, JCM19995, and CECT7894 have been deposited at DDBJ/ENA/GenBank under the accession numbers RJJN00000000, WJRJ00000000, and WKJF00000000, respectively.

### 2.3. Genomic and Pangenomic Analyses

Bifidobacterium longum genomes were downloaded from the National Center for Biotechnology Information GenBank database (https://ncbi.nlm.nih.gov/genbank) and the Department of Energy’s Joint Genome Institute Integrated Microbial Genomes and Microbiomes database (https://img.jgi.doe.gov) (see Table S1) [32,33]. For genomes found in both databases, the GenBank data were used. RAST and PROKKA v.1.13 were used for annotating genes using default parameters [29,30,31]. Pangenomes were calculated via Roary v.3.12.0 with the arguments -e and -n using the PROKKA gff files as input [34]. Gene content differences between subspecies were determined using the Roary query_pan_genome -a script. The Roary output was visualized using roary_plots.py and create_pan_genome_plots.R. Average nucleotide identity of all B. longum strains versus each other was calculated with pyani v.0.2.8 using the ANIm MuMmer method and plotted using heatmaps.2 in gplots [35,36].

*B. infantis* genomes were queried for HMO gene cluster genes as defined in *B. infantis* ATCC15697^T^ using BLAST [37]. The resultant genes were aligned by CLUSTALW using slow/accurate parameters [38]. Phylogenetic analysis was performed via the raxmlGUI v1.5 using maximum likelihood with 1000 bootstraps [39]. Phylogenetic trees were visualized and edited for clarity using FigTree v1.4.4 [40]. 

Pangenome gene sequences were provided in the pan_genome_reference.fa output file in Roary. These sequences were inputted into the EggNOG 4.5.1 eggNOG-mapper v2 genome-wide functional annotation tool, which was run with DIAMOND mapping mode and all other settings as default [41,42]. The results from EggNOG were downloaded as csv files and organized based on Clusters of Orthologous Groups (COGs) found in each individual genome or subspecies pangenome. Values associated with COG categories represent the percentage of COGs belonging to each category out of the total number of identified COGs. If a gene was assigned to two COG categories, each COG category was counted separately. When applicable, genes in the *B. infantis* pangenome categorized by PROKKA/Roary as hypothetical were further annotated using HMMER against the Pfam database [43,44]. Genes associated with carbohydrate metabolism were identified using the Carbohydrate-Active Enzymes (CAZy) database (http://www.cazy.org/) via the Database for Automated Carbohydrate-Active Enzyme Annotation (dbCAN) meta server [45,46]. CAZY hits were considered positive if they were identified by at least two of the three available annotation tools (HMMER v3.2, DIAMOND v0.9, and Hotpep v1). Gene gain and loss were determined using Count with default parameters, automated rate optimization, and a Dollo parsimony analysis [47]. Ecotype modeling was calculated based on the approach described by Konstantinos and Tiedje with modifications [48]. The average nucleotide identity (ANI) of each genome was calculated in reference to the type strain of each subspecies [35,48,49]. This value was then plotted against the average percent identity of protein-coding genes, also in reference to the subspecies type strain [48]. 

### 2.4. Species-Wide Phylogenetic Inference

Phylogenetic analyses were performed using the up-to-date bacterial core gene (UBCG) pipeline, which infers a maximum-likelihood phylogeny using the concatenated sequences of 92 single-copy core genes [50]. UBCG utilizes the external programs Prodigal v2.6.3, hmmsearch v3.2.1, MAFFT v7.313, and RaxML v8.2 in the pipeline [51,52,53,54]. Support for each node is calculated as a Gene Support Index (GSI) representing the number of genes (from a total of 92) whose common sequences group the taxa together within a branch. The *Bifidobacterium breve* DSM20213 genome was used as an outgroup and obtained from the NCBI GenBank accession GCA_001025175.1. Phylogenetic trees were visualized and formatted using FigTree v.1.4.4 [40].

### 2.5. Carbohydrate Fermentation Phenotyping

Bifidobacterial strains were evaluated for their ability to utilize carbohydrates as a sole carbon source. Briefly, 1% *v*/*v* overnight culture was grown in triplicate on modified MRS (mMRS) media containing 2% *w*/*v* of each carbon substrate: arabinose, fructo-oligosaccharides (FOS), fructose, glucose, lactose, mannose, melezitose, N-acetylglucosamine, raffinose, and xylose. Biomass production was estimated by measuring the optical density at 600_nm_ (OD_600_). To determine final OD_600_, mMRS was inoculated with an overnight culture at a concentration of 1% and then incubated for 72 h at 37 °C under anaerobic conditions. Two-way analysis of variance was performed using GraphPad Prism version 6 (GraphPad Software, La Jolla, CA, USA). In addition, isolates were tested for growth on lacto-N-tetraose (LNT) and lacto-N-neotetraose (LNnT) using a PowerWave HT Microplate Spectrophotometer (BioTek, Winooski, VT, USA). Overnight cultures grown in MRS were used to inoculate mMRS at a concentration of 1%. Isolates then grew anaerobically for 40 h at 37 °C with shaking and OD_600_ measurements every 15 min. The OD_600_ values were plotted using GraphPad Prism 6. 

## 3. Results

### 3.1. Bifidobacterium longum General Genome Characteristics

**Figure 1:**
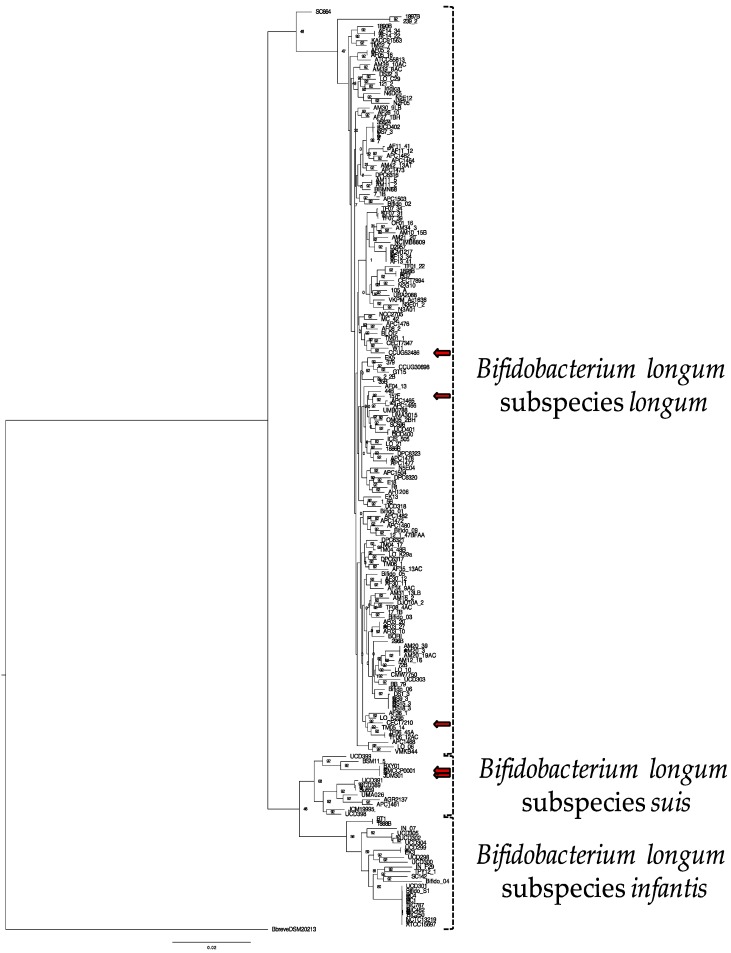
Phylogenetic tree of 191 *B. longum* strains using the concatenated sequences of 92 core single-copy genes via the UBCG pipeline. Node labels indicate the Gene Support Index value which represents the number of genes (from a total of 92) whose common sequences group the taxa together within a branch. Strains with assigned subspecies in public databases that are inconsistent with this phylogenetic analysis are shown with red arrows (157F, CECT7210, CCUG52486, JDM301, CMCCP0001). *Bifidobacterium breve* DSM20213 is used as an outgroup.

A total of 191 *Bifidobacterium longum* genomes were analyzed (Supplemental Table S1). Two of these strains, UMA3015 and JCM19995, had their genomes sequence de novo, with the former isolated from infant feces and the latter isolated from pig feces [55]. The genome of a third strain, CECT7894, was provided by AB-Biotics and has been deposited in the NCBI GenBank database. Of 191 genomes, 71 (37.2%) had been assigned a subspecies designation within a public database: *longum* (43), *infantis* (22), and *suis* (6) (Supplemental Table S1). Noteworthy, 5 of the 71 strains (i.e., 157F, CECT7210, CCUG52486, JDM301, CMCCP0001) possess a subspecies designation that conflicts with our phylogenetic analysis (see Figure 1 and Supplemental Table S1). The misidentification of these strains has been noted in previous reports and is a result of challenges in accurately typing the closely related *B. longum* subspecies [14,18,56]. The average genome size for all *B. longum* strains is 2.42 Mb with a minimum of 1.87 Mb (strain 121.2) and a maximum of 2.88 Mb (strain BIC1307292462) (Table 1 and Supplemental Table S1). The average genome size of the *infantis* subspecies (2.67 Mb) is significantly larger than *longum* (2.38 Mb) and *suis* (2.42 Mb) (Table 1). The percent GC content of each subspecies does not vary considerably (subspecies *longum*, 60.03%, subspecies *infantis*, 59.70%, and subspecies *suis*, 59.85%). Interestingly, the mean genome size of 9 *B. infantis* strains (ATCC15697, BIC1206122787, BIC1307292462, BIC1401111250, Bifido_S1, NCTC13219, PC1, PC4, and UCD301) is significantly higher than the mean genome size of the remaining 14 *B. infantis* strains (2.78 ± 0.08 versus 2.59 ± 0.09; *p* < 0.01 via unpaired *t* test).

### 3.2. Bifidobacterium longum Inferred Phylogeny

The evolutionary relationship between *B. longum* strains was inferred using the Up-to-Date Bacterial Core Gene (UBCG) pipeline via EZBioCloud, which uses the concatenated sequences of 92 single-copy core genes [50]. These core genes represent conserved phylogenetically informative loci that exhibit consistent divergence rates and are not likely a product of horizontal transfer. Maximum likelihood was applied to construct the resultant tree (Figure 1). The topology of this phylogeny indicates the separation of the *B. longum* species into the three accepted subspecies, with most strains segregating to *longum* and *suis*/*infantis* assembling into a distinct clade. This reflects subspecies divergence from a common ancestor. Furthermore, topology of this *B. longum* phylogeny is similar to a recent study of several *Bifidobacterium* species [57]. Within the *infantis* subspecies, there is a clear delineation between the strains where some cluster tightly with the remainder exhibiting more diversity. Interestingly, this clustering pattern matches the genome size disparity observed among *infantis* strains. Strain *B. longum* JCM19995 was proposed to be in a distinct and fourth subspecies closely related to subspecies *suis*, termed subspecies *suillum*, based partially on its urease-negative phenotype [55]. Interestingly, JCM19995 shares a node with *B. suis* UCD398, a urease-negative strain, but does not segregate with other strains lacking the urease gene cluster (Figure S1). This does not support assigning *B. longum* JCM19995 to a novel subspecies; rather, it is one of several *B. longum* subspecies *suis* strains lacking urease activity.

### 3.3. Average Nucleotide Identity Analyses

**Figure 2:**
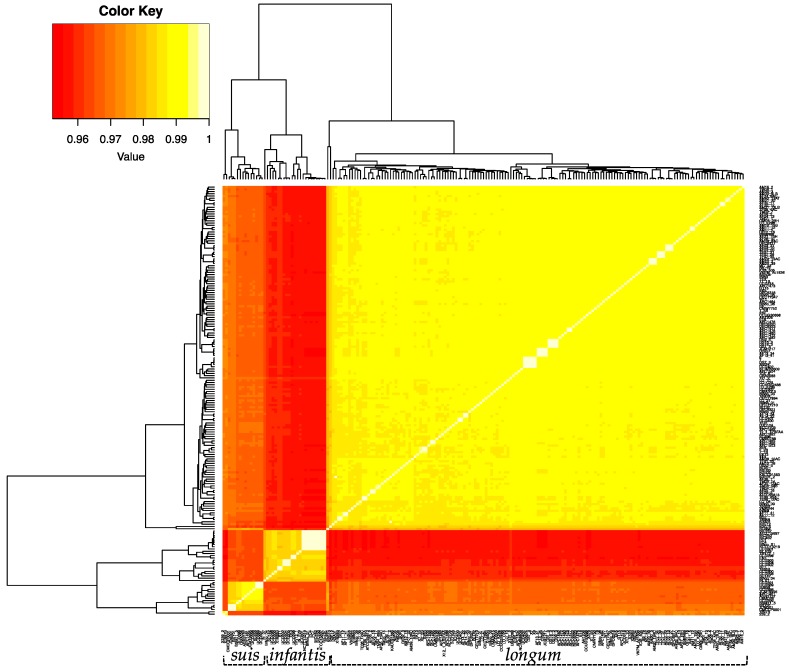
Heatmap displaying the percent average nucleotide identity (ANI) between the 191 *B. longum* strains. ANI was calculated via pyani v.0.2.8. using the ANI MUMmer/NUCmer method. The color key represents the percentage identity of strains with lower (red) and higher (yellow) ANI values. Strains clustered via dendrograms based on row means with gplots heatmap.2.

**Figure 3:**
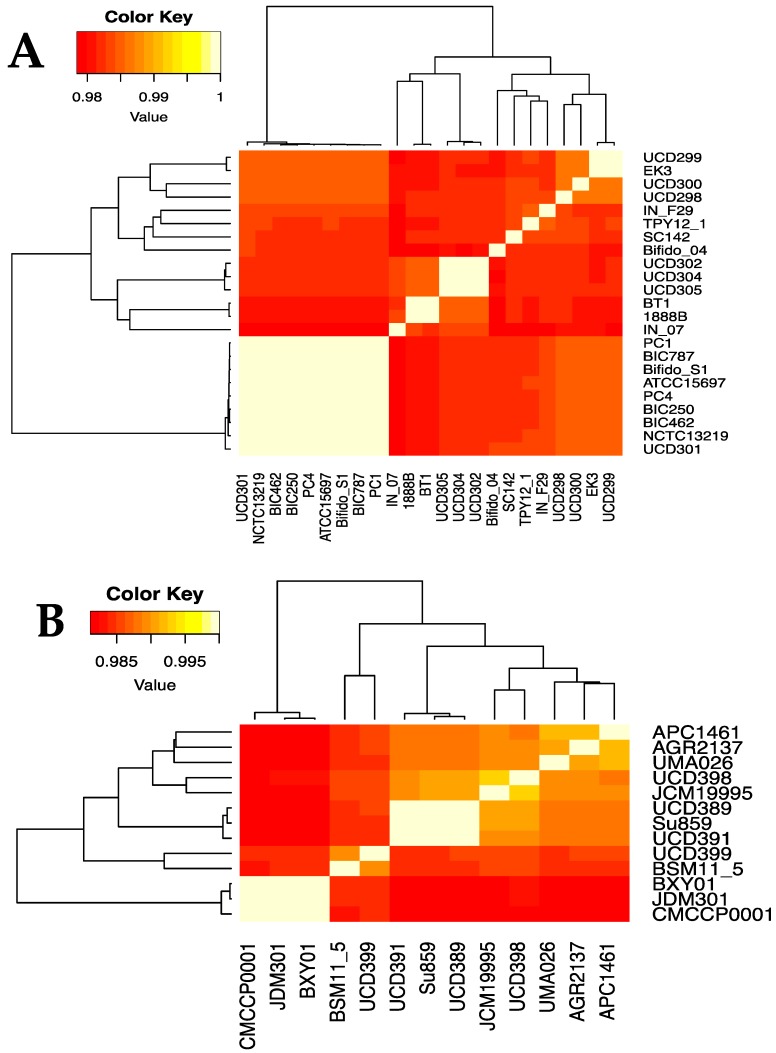
Heatmap indicating the percent average nucleotide identity (ANI) between the 23 *B. infantis* (**A**) and 13 *B. suis* (**B**) strains. ANI was calculated via pyani v.0.2.8. using the ANI MUMmer/NUCmer (ANIm) method. The color key represents the percentage identity of strains with lower (red) and higher (yellow) ANI values. Strains clustered via dendrograms based on row means with gplots heatmap.2.

In order to further define the genomic relationships between the three subspecies, we calculated the percent average nucleotide identity (ANI) of all genomes against each other (Figure 2 and Supplemental Table S2). The ANI values of the 191 *B. longum* strains cluster into three groups corresponding to the three subspecies and recapitulate the single-copy gene phylogeny (Figure 1). Subspecies *longum* has the widest ANI range, with several strain/strain ANI values below 97%. Strain-level detail of ANI similarities between *infantis* and *suis* strains is depicted in Figure 3A,B. Taken together, the UBCG phylogeny and the ANI analysis support the separation of *B. longum* species strains into three separate subspecies. In addition, the separation of *infantis* strains into two groups in the inferred phylogeny and the ANI analysis demonstrates significant subspecies variation. It is possible that the *B. infantis* strains closely related to ATCC15697 are derived from this source strain and used in probiotic products. In support of this, several of these strains were isolated from the blood of patients taking oral probiotics [58,59]. 

**Figure 4:**
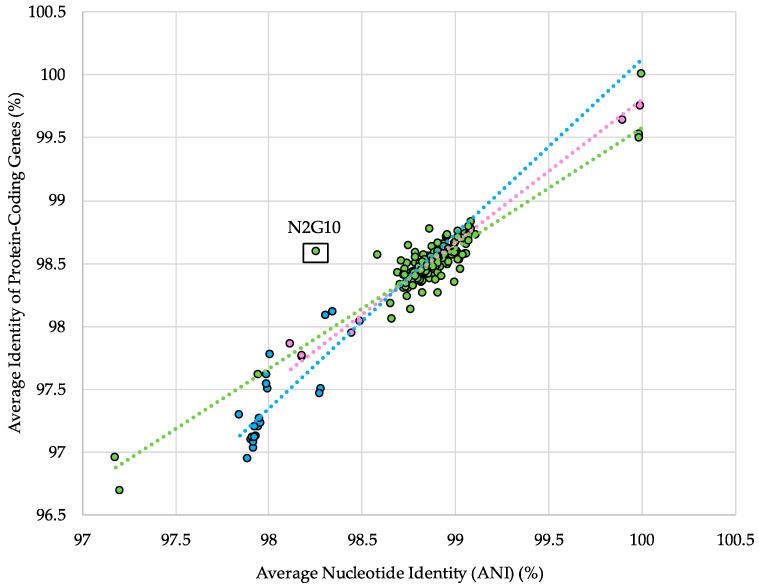
Percent average nucleotide identity (ANI) versus the percent average nucleotide identity of protein-coding genes, both calculated for each individual strain against a representative genome of its subspecies. Colored dots represent the strains from subspecies *longum* (green), *infantis* (blue), and *suis* (pink). Dotted lines represent the lines of best fit for each individual subspecies dataset.

Despite the complementary and synergistic power of the ANI comparison and the concatenated core gene phylogeny in distinguishing subspecies, these analyses do not account for functional differences. To determine the relationship with predicted gene content, the ANI between each strain and a representative strain from its subspecies was plotted against the ANI of protein-coding genes (ANI_pcg_). Under neutral conditions, one would expect a linear relationship between changes in ANI and ANI_pcg_, and variations in this pattern may suggest differences in ecotypes within a subspecies. The results of this analysis identified one major ecotype outlier, *B. longum* subspecies *longum* N2G10 (Figure 4). It is unclear what factors may have influenced the high ANI_pcg_ of this strain relative to its ANI. However, this strain is one of eight isolated from vaginal swab samples, a rare isolation source among *B. longum* genomes, and the genome assembly contains a high number of contigs (Supplemental Table S1) [60].

### 3.4. The Bifidobacterium longum Pangenome

**Figure 5:**
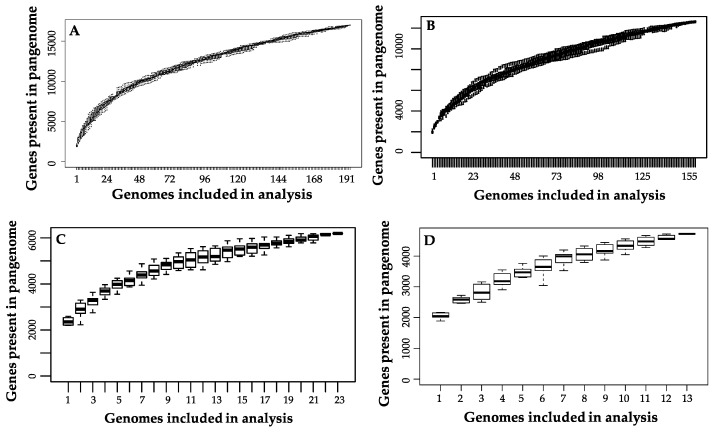
Pangenome plots for (**A**) all *B. longum* strains, (**B**) *longum* subspecies, (**C**) *infantis* subspecies, and (**D**) *suis* subspecies.

**Figure 6:**
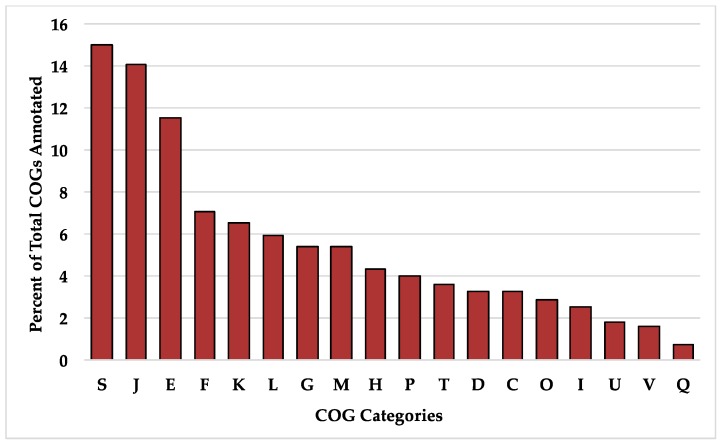
Percent of total clusters of orthologous groups (COGs) annotated in the *B. longum* core gene set. Single letter COG category designations are as follows: S, function unknown; J, translation, ribosomal structure, and biogenesis; E, amino acid transport and metabolism; F, nucleotide transport and metabolism; K, transcription; L, replication, recombination, and repair; G, carbohydrate transport and metabolism; M, cell wall/membrane/envelope biogenesis; H, coenzyme transport and metabolism; P, inorganic ion transport and metabolism; T, signal transduction mechanisms; D, cell cycle control, cell division, and chromosome partitioning; C, energy production and conversion; O, post-translational modification, protein turnover, and chaperones; I, lipid transport and metabolism; U, intracellular trafficking, secretion, and vesicular transport; V, defense mechanisms; Q, secondary metabolite biosynthesis, transport, and catabolism.

The *B. longum* species pangenome is open but is approaching a closed state given the current availability of strains in public databases. This is consistent with prior analyses conducted on *B. longum* [19,20]. Thus additional strains are required to describe the full diversity potential (Figure 5A). The *B. longum* pangenome contains 16,973 total genes, a majority of which (85.2%) are found in ≤28 strains. Out of the total gene set, only 551 (3.2%) are core genes defined as present in ≥190 strains (Table 2). The majority of identified COGs in the core gene set were assigned to the S category with an unknown function (15.0%), J which encompasses housekeeping functions translation, ribosomal structure, and biogenesis (14.1%), and E which includes amino acid transport and metabolism (11.6%) (Figure 6). The high prevalence of COG categories J and E represents cellular functions whose metabolic pathways are conserved among *B. longum* strains, whereas categories with less core gene representation demonstrate more strain-level metabolic flexibility. There are 124 gene sequences (22.5% of total core genes) annotated as “hypothetical protein”, “putative protein”, or “putative membrane protein”. Other genes included in the core set are essential to housekeeping functions, including cell division (*ftsL*, *ftsQ*, *ftsZ*, *crgA*, and *sepF*), protease activity (*clpP* and *clpX*), and peptidoglycan synthesis (*pbpA*, *pbpB*, and *pbpG*). Several core genes are known to interact with carbohydrates that *B. longum* encounter in the gut, including β-N-acetylhexosaminidase (EC 3.2.1.52), a glycosyl hydrolase critical for metabolizing human milk oligosaccharides, levanase (EC 3.2.1.65), which facilitates hydrolysis of fructose polymers, and various ABC transporters [61,62]. As expected, all genomes encode the bifidobacterial characteristic enzyme fructose-6-phosphate phosphoketolase (EC 4.1.2.22) which is critical in the central fermentative pathway termed the bifid shunt [63].

**Figure 7:**
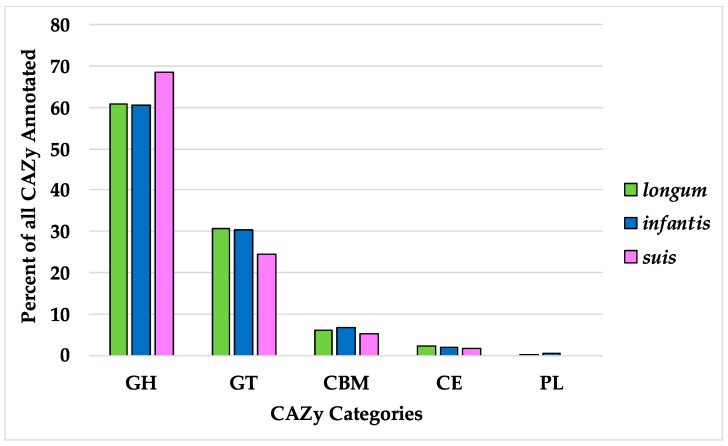
Percent of all CAZy categories identified in the three subspecies pangenomes. CAZy category definitions are as follows: GH, glycoside hydrolases; GT, glycosyl transferases; CBM, carbohydrate-binding modules; CE, carbohydrate esterases; and PL, polysaccharide lyases.

Bifidobacterial metabolism is primarily understood from its capacity to ferment carbohydrates. Thus carbohydrate-active enzyme (CAZy) gene homologs were identified in *B. longum* to define the distribution of this important class of genes [45,46]. By convention, carbohydrate genes are sorted as glycoside hydrolases (GH), glycosyltransferases (GT), polysaccharide lyases (PL), carbohydrate esterases (CE), and carbohydrate-binding modules (CBM). Of the *B. longum* core genes, nine were identified as carbohydrate-active enzymes including five GHs and four GTs (Table 3). Among these was GH13, a CAZy associated with plant-based carbohydrates which Milani et al. identified as being the most common CAZy in bifidobacterial genomes [64]. GHs were identified as the highest fraction of total CAZys, with the *B. suis* pangenome exhibiting the greatest proportion of the three subspecies (Figure 7). Interestingly, a broader pangenomic analysis identified almost identical proportions of GH (43.4%) and GT (43.8%) in the family *Bifidobacteriaceae* as a whole [65]. The high proportion of GH versus other carbohydrate-active enzymes in *B. longum* reflects their critical role in processing plant- and host-derived carbohydrates.

**Figure 8:**
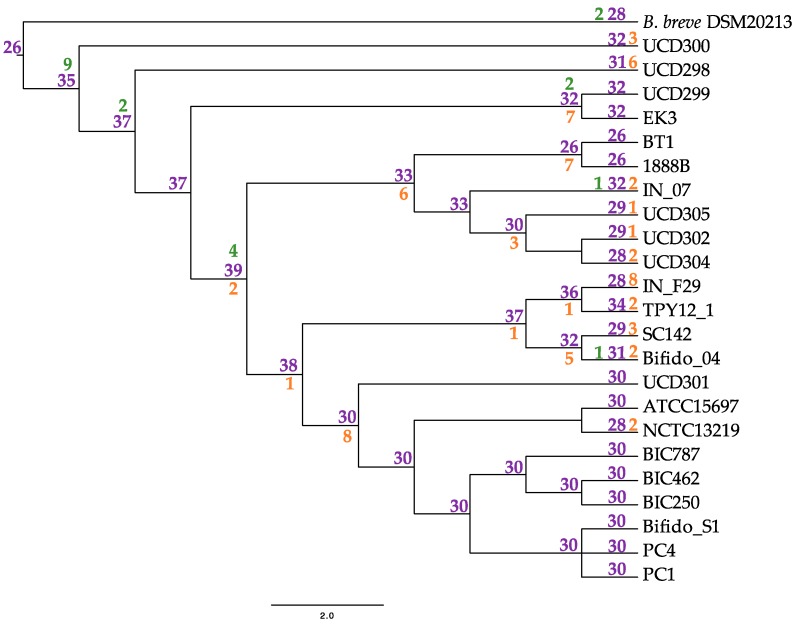
Inferred glycosyl hydrolase gene flux in extant *Bifidobacterium longum* subspecies *infantis*. Nodes are represented in purple with gene gain denoted in green and loss in orange.

Each subspecies pangenome possesses at least one GH not present in the others. Subspecies *longum* and *suis* both contain GHs postulated to interact with plant-associated carbohydrate substrates. This includes GH16 (xyloglucan metabolism) in subspecies *suis* and GH65 (EC 3.2.1.28) in subspecies *longum*, which we postulate is linked to the plant-based diet of their host (Table 4) [66,67,68]. Subspecies *infantis* strains colonize the nursing infant gut, thus they encode an assortment of HMO metabolic enzymes within their genome. Accordingly, the *infantis* subspecies possess the HMO-degrading enzyme GH151 α-L-fucosidase (EC 3.2.1.51) and two GHs assigned N-acetylgalactosaminidase functions (GH109; EC 3.2.1.49) and (GH123; EC 3.2.1.53) (Table 4) [68,69,70,71]. Glycosyl hydrolases are essential for *B. infantis* HMO utilization, thus the gain and loss pattern of GHs within *B. infantis* was mapped onto the *B. infantis* phylogeny. This analysis infers 35 GH in the common ancestor of the *infantis* subspecies, followed by gain and loss events at 6 and 21 nodes, respectively (Figure 8). Numbers of GH in extant *infantis* strains varied from 26 (BT1, 1888B) to 34 (TPY12_1).

### 3.5. Comparative Pangenomics between Bifidobacterium longum Subspecies

**Figure 9:**
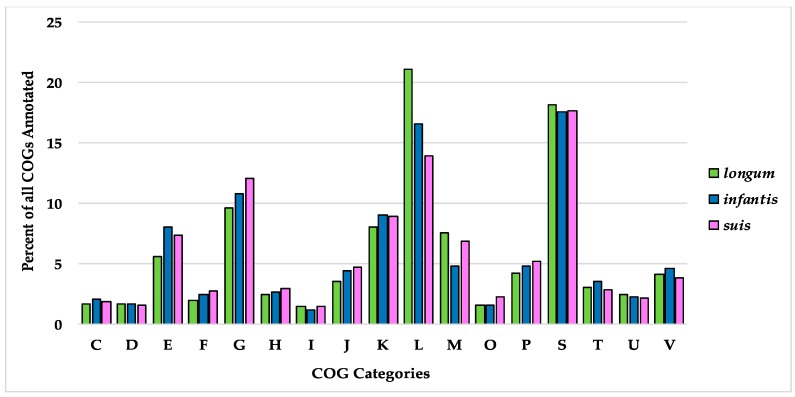
Distribution of Clusters of Orthologous Groups of proteins (COGs) identified in the three subspecies pangenomes. Single-letter COG category designations are as follows: C, energy production and conversion; D, cell cycle control, cell division, and chromosome partitioning; E, amino acid transport and metabolism; F, nucleotide transport and metabolism; G, carbohydrate transport and metabolism; H, coenzyme transport and metabolism; I, lipid transport and metabolism; J, translation, ribosomal structure, and biogenesis; K, transcription; L, replication, recombination, and repair; M, cell wall/membrane/envelope biogenesis; O, post-translational modification, protein turnover, and chaperones; P, inorganic ion transport and metabolism; S, function unknown; T, signal transduction mechanisms; U, intracellular trafficking, secretion, and vesicular transport; V, defense mechanisms.

Among 155 subspecies *longum* strains, 761 (6.0%) core genes shared (≥154 strains) were identified. Most genes of the *longum* subspecies pangenome (10,278; 81.5%) are only found in ≤23 strains (Table 2). Of the three subspecies, *longum* strains have the lowest percentage of core genes despite their close relatedness as determined by phylogenetics and ANI. In this current analysis, the *longum* subspecies pangenome is approaching but has not reached an asymptote, similar to a 2015 analysis performed by Chaplin et al. [18]. Within the subspecies *longum* pangenome, the highest represented COG categories are S: function unknown (20.40%), L: replication, recombination, and repair (21.48%), and G: carbohydrate transport and metabolism (10.72%). In comparison to the other subspecies, *longum* has the greatest proportion of COGs associated with replication, recombination, and repair (COG category L), cell wall/membrane/envelope biogenesis (COG category M), and defense mechanisms (COG category V) (Figure 9). Subspecies *longum* uniquely contains genes belonging to the COG category W (extracellular structures). Two genes COG category W are predicted collagen triple helix repeat protein and found in strain 12_1_47BFAA. An additional COG W gene, collagen type I alpha 1, was unique to strain APC1476. Collagen-related proteins are associated with the mammalian cell extracellular matrix, with some host-associated bacteria expressing collagen-like proteins to interact with host cells [72].

Thirteen *B. longum* strains were assigned to subspecies *suis* through phylogenetic relatedness and ANI. This includes strain JCM19995 which was previously proposed to belong to a fourth subspecies *suillum,* distinct from subspecies *suis*. The evidence presented in support of this taxonomic reorganization includes amplified fragment length polymorphisms, multilocus sequence analysis, multilocus sequence typing, and urease activity assays [55]. The lack of urease activity contributed prominently in the designation of a novel subspecies, although the original description of subspecies *suis* indicates variability in urease activity [7]. Thus urease activity is not a reliable discriminatory parameter for membership in subspecies *suis*. Accordingly, 6 of 13 subspecies *suis* genomes lacked all urease cluster genes: JCM19995, JDM301, CMCCP0001, BXY01, UCD398, and UMA026 (Supplemental Figure S1). Furthermore, there were no genes identified to all non-urease strains not found in the other *B. suis* strains. Therefore, the current analyses did not provide evidence to segregate JCM19995 from other *suis* strains, therefore not supporting subspecies *suillum* as a usable taxonomic boundary as currently defined (Figure 1 and Figure 3B). 

Overall, the subspecies *suis* pangenome contains 4723 genes, of which 1187 (25.13%) are core genes (Table 2). As with subspecies *longum,* subspecies *suis* possesses a high representation of COG categories S: function unknown (20.26%), L: replication, recombination, and repair (14.47%), and G: carbohydrate transport and metabolism (12.76%) (Figure 9). In addition, the subspecies *suis* pangenome contains a higher percentage COGs associated with G: carbohydrate transport and metabolism, O: post-translational modifications, protein turnover, and chaperones, and P: inorganic ion transport and metabolism than subspecies *longum* and *infantis*. The subspecies *suis* pangenome is open, although it appears close to saturation (Figure 5C).

The subspecies *infantis* pangenome contains 6196 genes, 1019 (16.4%) of which are core genes. Almost half of the total genes (2980; 48.1%) are present in a maximum of three *B. infantis* strains (Table 2). Out of all the genes, 3998 (64.5%) were labeled as hypothetical, which is consistent with a prior study noting the high amount of hypothetical genes in the full subspecies *infantis* pangenome [18]. The subspecies *infantis* pangenome plot suggests that genomic diversity did not reach saturation as visualized as an asymptote (Figure 5D). As with the *suis* pangenome, the most highly represented COG categories are function unknown (COG category S; 20.88%), replication, recombination, and repair (COG category L; 18.17%), and carbohydrate transport and metabolism (COG category G; 11.71%). Overall, the representation of COGs is more evenly distributed across the COG categories in subspecies *infantis* versus subspecies *longum* and subspecies *suis* (Figure 9).

### 3.6. Variation within the Bifidobacterium longum Subsp. Infantis HMO Gene Cluster

**Figure 10:**
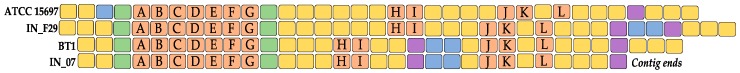
The human milk oligosaccharide (HMO) utilization cluster in *B. infantis* strains ATCC15697, IN_F29, BT1, and IN_07. Genes are represented by blocks color-coded according to their function: yellow, carbohydrate transporters; blue, transposable elements; green, hypothetical genes; orange, HMO utilization enzymes; purple, uracil-related genes. Letters correspond to the HMO utilization enzymes listed in Table 5.

*B. infantis* strains are characterized by the presence of a ~40 Kb gene cluster associated with HMO utilization within their genomes [15]. This region has been shown to contain a combination of HMO-active enzymes, including α-L-fucosidase (EC 3.2.1.51), exo-α-sialidase (EC 3.2.118), other glycosyl hydrolases, and ABC transporters to translocate HMOs intracellularly [14,15,73]. There is significant scientific evidence that *B. infantis* utilizes milk components as an evolved nutritive strategy, thus it is unsurprising that the HMO gene cluster is generally conserved across strains. Of the *B. infantis* genomes available, four strains with single-contig HMO clusters were selected for further analysis (ATCC 15697^T^, IN_07, IN_F29, and BT1). All clusters encode several anchor features, including 12 carbohydrate-related enzymes and oligosaccharide permeases (e.g., ABC transporters), in addition to mobile elements and hypothetical proteins (Figure 10 and Table 5). The cluster was originally described in Sela et al. 2008, and is defined herein as beginning with two major facilitator superfamily membrane transport proteins (Blon_2331 and Blon_2332) to terminate with a carbohydrate ABC transporter membrane protein (Blon_2369), with carbohydrate-related enzymes and transporter components interspersed between [15]. 

**Figure 11:**
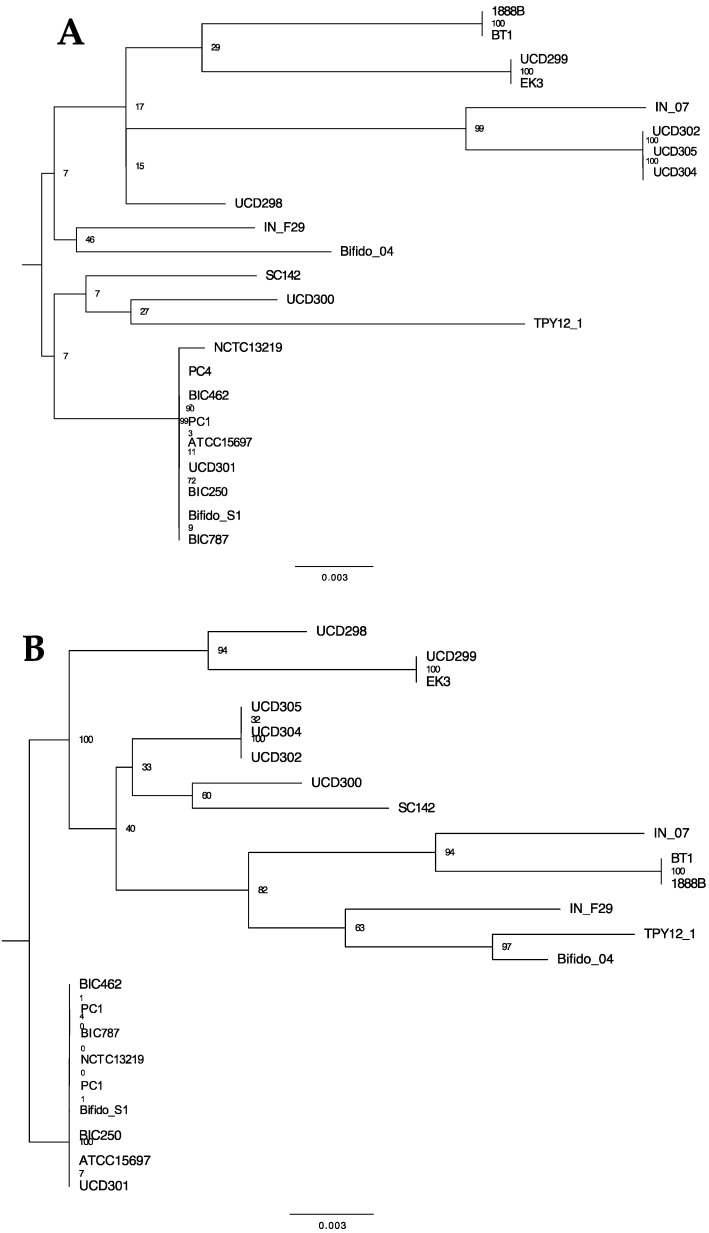

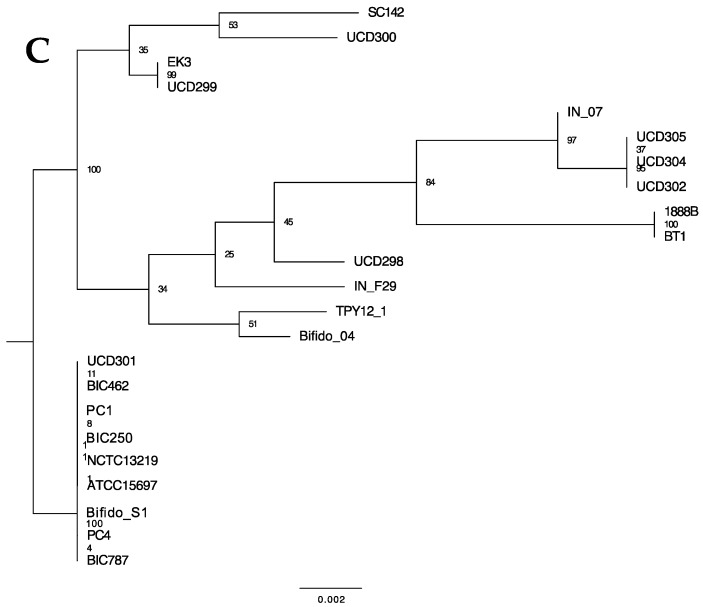
Phylogenetic relationships between HMO cluster glycosyl hydrolases (**A**) sialidase, (**B**) α-fucosidase GH95, and (**C**) α-fucosidase GH29 gene sequences with 1000 bootstrap replicates. Node labels indicate the Gene Support Index value which represents the number of genes (from a total of 92) whose common sequences group the taxa together within a branch.

The HMO utilization gene cluster was first identified in *B. longum* subspecies *infantis* ATCC15697^T^ and subsequently determined to be present in all subspecies *infantis* strains examined to date [14]. There are six carbohydrate transporter genes within the ATCC15697 gene cluster (Blon_2342 to Blon_2347) and conserved in IN_F29. It is likely that these arose from a duplication event of three genes, two ABC permeases and their associated extracellular solute-binding protein. Strains IN_07 and BT1 do not exhibit this duplication event which may represent a distinct lineage. These two strains, however, exhibit a likely duplication where additional copies of genes adjacent to the HMO cluster have been inserted within the cluster proximal to two transposases (Figure 10). Interestingly, these duplicated genes are not predicted to be involved directly in HMO catabolism. Maintenance of cluster integrity (i.e., colinearity) is measured by presence/absence and sequential order of genes. Moreover, the sequence of individual HMO-active enzymes reflects evolutionary divergence potentially consistent with phylogenetic divergence of the strains. To this end, three HMO-active genes were further analyzed to catalog variation and infer phylogenetic relatedness. The GH29 fucosidase (Blon_2336) and GH95 fucosidase (Blon_2335) contain nonsynonymous mutations at several sites along the genes which may reflect differences in HMO utilization phenotypes between groups. Both c gene trees depict the same nine strains separating into their own branch at the root of the tree (Figure 11B,C). These nine strains form also a distinct group in the genome-based phylogeny, but in that phylogeny, the nine strains share a node with IN_F29, TPY12_1, SC142, and Bifido_04 (Figure 1). The gene tree representing glycosyl hydrolase exo-α-sialidase (Blon_2348) is consistent with the genome-based phylogeny for many branches, although the exo-α-sialidase tree shows UCD300 as being more closely related to ATCC15697.

### 3.7. Bifidobacterium longum Carbohydrate Metabolism Phenotypes

**Figure 12:**
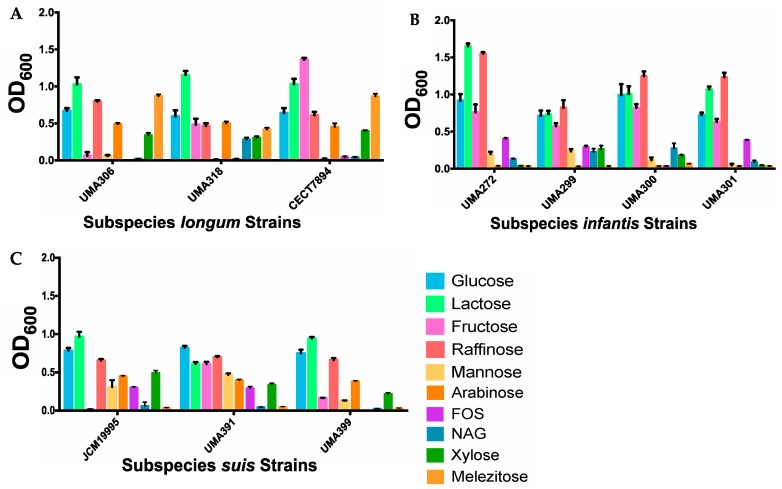
*Bifidobacterium longum* strains generate biomass from utilizing sole carbohydrate sources inferred from optical density at 600_nm_. Panels (**A**) *longum*, (**B**) *infantis*, and (**C**) *suis* grown on sole carbohydrates.

Although each subspecies generally exhibits a common genomic profile, individual strains may exhibit variant phenotypes under similar in vitro conditions. Thus, representative strains were evaluated for their ability to grow on sole carbohydrate sources. Substrates tested included arabinose, fructo-oligosaccharides (FOS), fructose, glucose, lactose, mannose, melezitose, N-acetylglucosamine (NAG), raffinose, and xylose. These substrates are known to be used by members of the genus *Bifidobacterium* as fermentable carbohydrates (Figure 12). *B. longum* subsp. *longum* strain CECT7894 grew significantly (*p* < 0.05) more than both UMA306 and UMA318 on fructose and xylose with mean OD_600_ values of 1.36 and 0.40, respectively. Interestingly, UMA306 grew the most on raffinose (mean OD_600_ = 0.80; *p* < 0.0001) as a sole carbohydrate source (Figure 12A). Previous studies indicated that specific strains of subspecies *longum* grow efficiently on raffinose [74]. The subspecies *longum* strains did not grow substantially on FOS, which is inconsistent with previous studies conducted on *longum* strains on this substrate [10,75,76]. This may be due to strain and media differences between studies. The subspecies *longum* strains did not grow on NAG. This result is congruous with another study documenting the inability of *B. longum* subsp. *longum* NCC2705 to grow on this substrate [77]. Studies characterizing subspecies *longum* growth on mannose indicate variability, even when analyzing the same strain (*B. longum* NCC2705) [77,78]. All *B. infantis* strains grew considerably on glucose (mean OD_600_ = 0.71–0.99), lactose (mean OD_600_ = 0.73–1.65), fructose (mean OD_600_ = 0.58–0.82), and raffinose (mean OD_600_ = 0.82–1.55) (Figure 12B). None of the *B. infantis* strains grew substantially on arabinose, which is consistent with previous reports [8]. Among *B. suis* strains, none grew appreciably on NAG or melezitose, while all grew on glucose, lactose, raffinose, arabinose, mannose, and xylose (Figure 12C). UMA391 utilized fructose most efficiently (mean OD_600_ = 0.61; *p* < 0.0001), whereas UMA399 grew to a less extent (mean OD_600_ = 0.16) and JCM19995 did not exhibit growth. It is noteworthy that strains of subspecies *longum* and *suis* grew on arabinose, whereas *infantis* strains did not. Strains assigned to subspecies *longum* utilized melezitose, while *infantis* and *suis* strains were incapable of doing so. These findings are consistent with long understood differentiating phenotypes between *B. longum* subspecies [7].

## 4. Discussion

*Bifidobacterium longum* metabolizes substrates in the gut (i.e., dietary and endogenous) and is integrated into physiological networks between microbiota and their host. This microorganism is commonly used as a probiotic to promote gut health. Greater scientific understanding of the molecular interactions between *B. longum* with their host has been enabled by the development of analytical and bioinformatic approaches. *B. longum* research may therefore yield novel targets for direct nutritional interventions and lifestyle modification. This, of course, includes optimized prebiotic and probiotic approaches to maximize quantifiable benefits to the consumer.

The *B. longum* pangenome described herein catalogs the genomic potential of the species through a comparative analysis of constitutive genomes. This enabled identification of *B. longum* core and accessory genes linked with groups of *B. longum* strains. More specifically, these strain groups are conventionally referred to as subspecies (i.e., *infantis*, *longum*, and *suis*). Subspecies is a taxonomic entity for which an accepted universal definition does not exist for bacteria, at least not in practical application beyond ad hoc definitions for a single species (e.g., *Bifidobacterium animalis*). As bacterial species boundaries are amorphous, subspecies demarcations are similarly prone to uncertainty with the isolation of strains exhibiting genotypic and phenotypic exceptions. 

The emphasis on, and limitations in defining, a subspecies-centered perspective of *B. longum* have resulted in some taxonomic confusion. Accordingly, there are multiple examples of incorrectly assigned designations, most notably between subspecies *longum* and *infantis*. As this is primarily a taxonomic challenge, the potential to confound is mitigated and does not reflect metabolic or ecological function, although the predictive power of a subspecies assignment depends on the phenotype of interest. Novel subspecies *infantis* strains are predicted to utilize HMOs, an attribute that has considerable scientific support, whereas subspecies *longum* and *suis* isolates may be viewed as unlikely to be efficient consumers of milk oligosaccharides. However, this assumption may be incorrect or incomplete, as novel isolates may not conform to this potentially transient understanding. 

As per current laboratory practice, assigning a strain to a subspecies is primarily based on identifying discriminatory genomic sequences. There are sequence principles that are currently considered to be characteristic of a subspecies. This includes the HMO cluster found in all *infantis* strains isolated to date, whereas other *B. longum* strains do not possess the contiguous locus in its entirety. One could conclude that the HMO cluster is unique to *infantis* and encodes fucosidase and sialidase enzymes that are critical to its lifestyle within the nursing infant gut. It is, however, incorrect that only subspecies *infantis* possesses fucosidase and sialidase genes. As with phenotype, genomic plasticity within *B. longum* may restrict firm subspecies definitions. 

Accordingly, evidence for the proposed subspecies *suillum* is based on limited sequencing and the absence of ureolytic activity. Support for the fourth subspecies is undermined by the limited number of candidate *suillum* strains evaluated, and the isolation of *suis* strains that lack urease genes and activity. Additional isolates are thus required to validate subspecies *suillum* and exclude the alternative hypothesis that these are subspecies *suis* strains. 

The three *B. longum* subspecies pangenomes are open and contain a considerable fraction of putative genes designated hypothetical or not assigned a function. The pangenomes of subspecies *infantis* and *suis* appear to approach saturation, suggesting that genetic accumulation primarily occurs in their core gene set. The subspecies *longum* pangenome reflects a tendency to utilize plant-based products inherent to the diet of their postweaned human host. This includes plant carbohydrate utilization genes such as amylopullanase and pectineasterase that are currently described as unique to subspecies *longum*, although this is subject to scientific revision as more strains are isolated. 

The genetic granularity by which *B. longum* is understood will continue to increase as more genome sequences are deposited into public databases. Challenges remain that are not unique to bifidobacterial genomic research, including generating quality sequences, assemblies, and consistent unsupervised annotation in the absence of manual curation. Thus establishing productive collaborative networks among bifidobacterial researchers could address these limitations through implementation of standardized genomic approaches. 

## Figures and Tables

**Table 1:** Average genome size (Mb) and average % GC content of the *B. longum* species and subspecies *longum*, *infantis*, and *suis*. Strains were assigned by phylogenetic and average nucleotide identity analyses.

Taxon	Average Genome Size (Mb)	Average % GC Content	Average No. of Genes	Substrate Preferences	Common Isolation Source
*Bifidobacterium longum* species	2.42	59.97	2155	Host-indigestible carbohydrates	Mammalian digestive tract
subspecies *longum*	2.38	60.03	2098	Plant-derived carbohydrate substrates	Human adults
subspecies *infantis*	2.67	59.70	2524	Human milk oligosaccharides (HMO)	Human infants
subspecies *suis* ^1^	2.42	59.85	2179	Plant-derived carbohydrate substrates	Nonhuman mammals

^1^ Includes *Bifidobacterium longum* JCM19995.

**Table 2:** Genes distributed across the *B. longum* species pangenome. Categories are defined as follows: core genes are identified in between 99% and 100% of strains; soft core genes are identified in between 95% and 99% of strains; shell genes are identified in between 15% and 95% of strains; cloud genes are identified in between 0% and 15% of strains.

Pangenome Scope	Core Genes	Soft Core Genes	Shell Genes	Cloud Genes	Total Genes
*Bifidobacterium longum* species	551	340	1613	14,469	16,973
subspecies *longum*	761	376	1194	10,278	12,609
subspecies *infantis*	1019	231	1966	2980	6196
subspecies *suis*	1187	0	1883	1653	4723

**Table 3:** *B. longum* CAZy domains identified in the core genes set.

Core CAZy Domain	Representative Gene ID	Inferred Function
GH3	*nagZ*	putative β-hexosaminidase
GH13	*glgE1*	α-1,4-glucan:maltose-1-phosphate maltosyltransferase
GH32	*sacA*	β-fructofuranosidase
GH36	*rafA*	α-galactosidase
GH77	*malQ*	4-α-glucanotransferase
GT2	*kfoC*	Putative glycosyltransferase
GT4	*mgtA*	glycosyltransferase
GT28	*murG*	UDP-N-acetylglucosamine--N-acetylmuramyl-(pentapeptide) pyrophosphoryl-undecaprenol N-acetylglucosamine transferase
GT51	*pbpG*	putative penicillin-binding protein

**Table 4:** Unique carbohydrate-active enzyme domains identified in *B. longum* subspecies pangenomes.

*Bifidobacterium longum* Subspecies	CAZy Domain	Representative Gene ID	Inferred Function
*longum*	CBM25	*group_8095* *	amylopullulanase
*longum*	CBM35	*hypBA2_4*	hypothetical protein
*longum*	CE8	*group_812* *	pectinesterase
*longum*	GH65	*kojP*	glycoside hydrolase family 65 protein
*infantis*	CBM5	*chiA*	carbohydrate-binding protein
*infantis*	CE2	*celE*	electron transport complex, RnfABCDGE type, D subunit
*infantis*	GH4	*licH*	glucosidase
*infantis*	GH151	*lacZ*	β-galactosidase
*suis*	GH16	*glcA*	β-galactosidase
*suis*	GH50	*group_2620* *	hypothetical protein
*suis*	GH59	*group_1820* *	carbohydrate binding family 6
*suis*	GH154	*group_1814* *	hypothetical protein

* Gene name not assigned.

**Table 5:** Select human milk oligosaccharide (HMO) gene cluster genes identified in *B. longum* subspecies *infantis* ATCC15697^T^. Locus tags and gene annotations are from the Joint Genome Institute Integrated Microbial Genomes and Microbiomes (IMG/M) database.

Gene Abbreviation Used in Figure 10	Locus Tag	Gene Annotation
A	Blon_2334	β-galactosidase
B	Blon_2335	α-L-fucosidase 2 (GH95)
C	Blon_2336	α-1,3/4-fucosidase (GH29)
D	Blon_2337	L-fucose mutarotase
E	Blon_2338	dihydrodipicolinate synthetase
F	Blon_2339	short-chain dehydrogenase/reductase SDR
G	Blon_2340	L-fuconate dehydratase
H	Blon_2348	exo-α -sialidase
I	Blon_2349	dihydrodipicolinate synthetase
J	Blon_2355	β-hexosaminidase
K	Blon_2356	haloacid dehalogenase domain protein hydrolase
L	Blon_2358	β-lactamase domain protein

## Supplementary Materials

The following are available online at https://www.mdpi.com/2076-2607/8/1/7/s1, Figure S1: Phylogenetic tree denoting urease-negative *B. suis* strains, Table S1: Strains included in this study, Table S2: Average nucleotide identity calculated for all strains.
