# Supplementary figures and images for: Comparative Pangenomics of the Mammalian Gut Commensal Bifidobacterium longum
Source: Microorganisms. 2019 Dec 18;8(1):7. doi: 10.3390/microorganisms8010007 (PMC7022738; doi:10.3390/microorganisms8010007)

## Slide 1
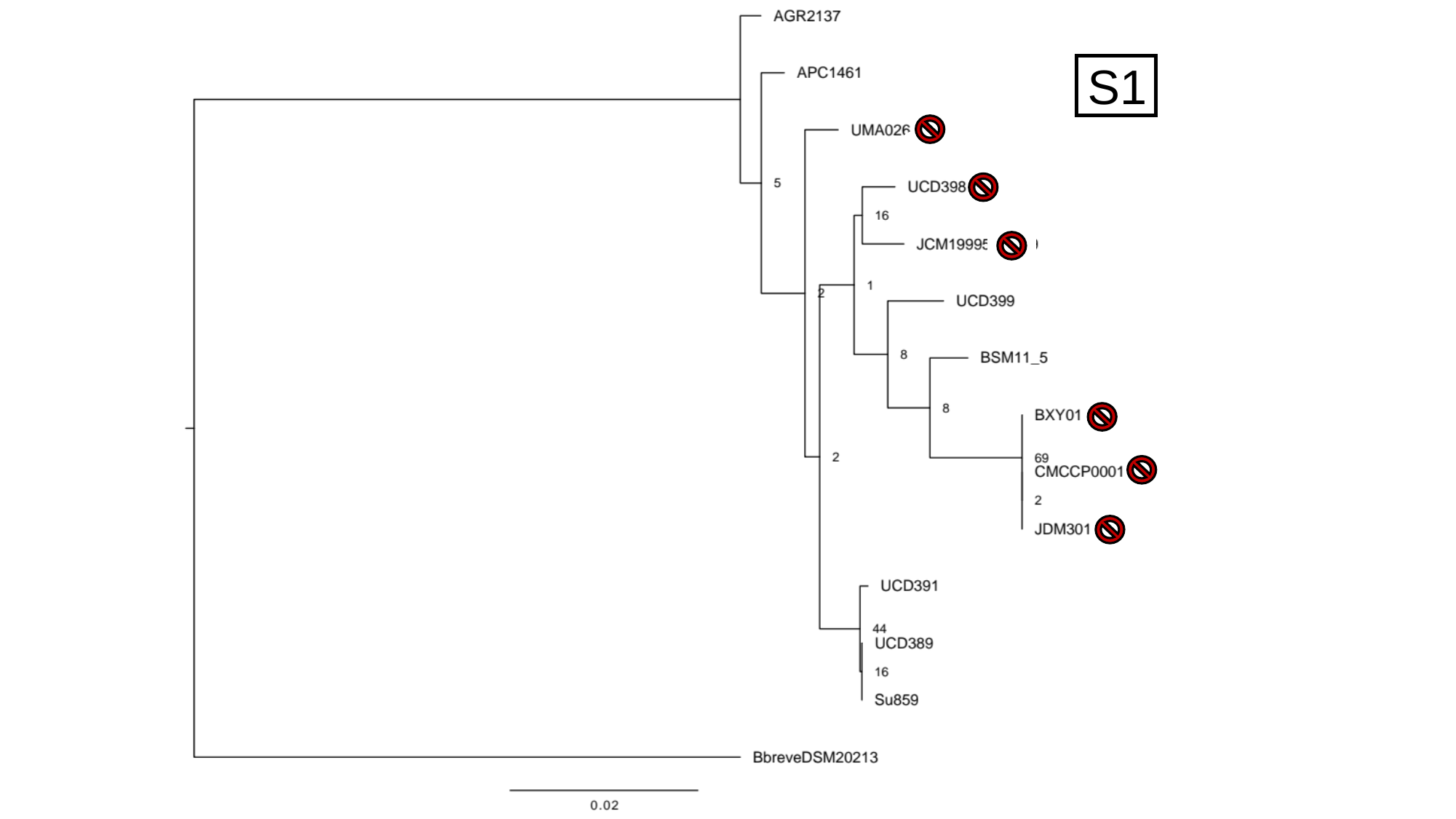

S1

Supplement: Supplementary file 1 [file microorganisms-08-00007-s001.zip › DASela_SuppFigure_MicroorganismsSubmission_Nov2019.pptx]
